# Conversion of lignin model compounds by *Pseudomonas putida* KT2440 and isolates from compost

**DOI:** 10.1007/s00253-017-8211-y

**Published:** 2017-03-15

**Authors:** Krithika Ravi, Javier García-Hidalgo, Marie F Gorwa-Grauslund, Gunnar Lidén

**Affiliations:** 1 Department of Chemical Engineering, Lund University, P.O. Box 124, SE-221 00 Lund, Sweden; 2 Department of Chemistry, Applied Microbiology, Lund University, P.O. Box 124, SE-221 00 Lund, Sweden

**Keywords:** Compost, *Pseudomonas*, Lignin, Aromatic compound conversion, Bacterial metabolism

## Abstract

Starting from mature vegetable compost, four bacterial strains were selected using a lignin-rich medium. 16S ribosomal RNA identification of the isolates showed high score similarity with *Pseudomonas* spp. for three out of four isolates. Further characterization of growth on mixtures of six selected lignin model compounds (vanillin, vanillate, 4-hydroxybenzoate, *p*-coumarate, benzoate, and ferulate) was carried out with three of the *Pseudomonas* isolates and in addition with the strain *Pseudomonas putida* KT2440 from a culture collection. The specific growth rates on benzoate, *p*-coumarate, and 4-hydroxybenzoate were considerably higher (0.26–0.27 h^−1^) than those on ferulate and vanillate (0.21 and 0.22 h^−1^), as were the uptake rates. There was no direct growth of *P. putida* KT2440 on vanillin, but instead, vanillin was rapidly converted into vanillate at a rate of 4.87 mmol (g_CDW_ h)^−1^ after which the accumulated vanillate was taken up. The growth curve reflected a diauxic growth when mixtures of the model compounds were used as carbon source. Vanillin, 4-hydroxybenzoate, and benzoate were preferentially consumed first, whereas ferulate was always the last substrate to be taken in. These results contribute to a better understanding of the aromatic metabolism of *P. putida* in terms of growth and uptake rates, which will be helpful for the utilization of these bacteria as cell factories for upgrading lignin-derived mixtures of aromatic molecules.

**Electronic supplementary material:**

The online version of this article (doi:10.1007/s00253-017-8211-y) contains supplementary material, which is available to authorized users.

## Introduction

Lignin, the second most abundant organic polymer on Earth, is a heterogeneous alkyl-aromatic biopolymer found as a significant constituent of lignocellulose in the plant cell wall (Ayyachamy et al. 2013). The current main use of technical lignin, which is produced in the pulp and paper industry, is as a fuel for producing process steam and electricity. There are also other uses for it, in particular lignosulfonates originating from the sulfite pulping process, but the predominant technical lignin, kraft lignin, is almost exclusively used as a fuel in the recovery boilers. In a widening biorefinery industry, in which more biomass-derived carbohydrates are also used for production of chemicals, a further valorization of lignin is an essential component. Increasing the value of lignin as a source of bio-based chemicals would enable new opportunities (Beckham et al. 2016; Camarero et al. 2014). In order to use lignin in this way, an initial depolymerization is required. A large number of approaches for lignin depolymerization, including thermochemical and enzymatic degradation, have been proposed (reviewed, e.g., by Rinaldi et al. 2016; Xu et al. 2014). Almost all of these depolymerization strategies result in a mixture of monomeric and oligomeric aromatic lignin-derived compounds. Utilization and conversion of these lignin-based aromatic compounds will pave the way for the production of various products in biorefineries (Abdelaziz et al. 2016; Ragauskas et al. 2014).

Microorganisms capable of catabolizing lignin-derived aromatics are essentially fungi and bacteria (Bugg et al. 2011a). The microbial degradation of native lignin by basidiomycetous fungi (white rot and brown rot) has been extensively scrutinized (Martínez et al. 2005). These fungi produce several extracellular peroxidases, laccases, and additional oxidative enzymes which generate aromatic radicals that break down the complex linkages present in lignin to compounds of lower molecular weight. However, these findings have not been transferred to a commercial process, partly because of the difficulties associated to fungal genetics and protein expression (Ahmad et al. 2011). The search for lignin-catabolizing bacteria has been made from several environments such as pulp and paper mills, soils with a rich biodiversity, decaying lignocellulosic materials, and in the guts of wood-eating insects (Suman et al. 2016). Several bacterial species which are able to break down lignin have been reported, and many have shown a great flexibility in the metabolism of lignin-related aromatic compounds (Bugg et al. 2011b). *Comamonas* sp. B-9, which was isolated from eroded bamboo slips, was found able to degrade kraft lignin to low molecular weight compounds and use these as the sole source of carbon (Chen et al. 2012). In another study, 140 bacterial strains were isolated from soil of a rainforest rich in biodiversity, and *Bacillus pumilus* as well as *Bacillus atrophaeus* were shown capable of degrading kraft lignin and lignin model dimers (Huang et al. 2013). *Amycolatopsis* sp., *Pseudomonas putida* strains, *Acinetobacter* ADP1, and *Rhodococcus jostii* were found able to depolymerize high molecular weight lignins and also catabolize a wide variety of low molecular weight lignin aromatics (Salvachúa et al. 2015). *Rhodococcus opacus* DSM 1069 and PD630 have been reported capable of converting lignin model compounds into triacylglycerols under nitrogen-limiting conditions (Kosa and Ragauskas 2012; Wei et al. 2015). DypB peroxidase was recently identified in *R. jostii* RHA1, shown to have lignin-degrading activity (Ahmad et al. 2011). Understanding the catabolic pathways of these organisms is central for developing processes for utilizing depolymerized lignin. The bacterium *Sphingobium* sp. SYK-6 (formerly known as *Sphingomonas paucimobilis* SYK-6) is one of the best studied lignin-degrading bacteria. This species can utilize a broad range of lignin aromatics, and its catabolic pathways have been thoroughly examined (Masai et al. 2014).

The objective of the present study was to screen for mesophilic, aerobic, culturable, and robust bacteria able to grow on aromatic compounds derived from lignin. For this purpose, a growth-based screening was made using samples from mature vegetable compost. This kind of compost is an interesting environment, in which the prolonged action of soil microorganisms has resulted in a range of aromatic compounds originating from lignin depolymerization, such as humic substances (Tuomela et al. 2000). Soil microbial communities in composts are able to assimilate or mineralize the carbohydrate and protein components of the organic matter easily, whereas lignin and its derived products are more recalcitrant and stable in the environment.

Microorganisms present in the compost samples were selected in the present study by enrichment culture, using kraft lignin and kraft pulping lignin-enriched streams. Some of the most prominent bacterial strains found were isolated and taxonomically identified using 16S ribosomal RNA (rRNA) sequencing. A second objective was to examine some of the isolated organisms in more detail with respect to its metabolic capability on lignin model compounds. Consequently, the growth on selected lignin model compounds was assessed for the isolates and one culture collection strain, *P. putida* KT2440. Six model compounds from the funneling catabolic pathways were used as carbon sources in the growth characterization: vanillin, vanillate, 4-hydroxybenzoate, *p*-coumarate, benzoate, and ferulate. The specific growth rates on these carbon sources as well as the conversion rates were determined in shake flask cultures. In addition, experiments were carried out using mixtures of the selected aromatic model compounds to assess the capacities for simultaneous uptake as well as multi-auxic growth patterns.

## Materials and methods

### Media and strains

All the experiments were carried out using M9 mineral media containing 6 g L^−1^ Na_2_HPO_4_, 3 g L^−1^ KH_2_PO_4_, 0.5 g L^−1^ NaCl, 1 g L^−1^ NH_4_Cl, 1 mM MgSO_4_, and 100 μM CaCl_2_ (Sambrook and Russell 2001) with 10 mL L^−1^ trace element solution (Pfennig and Lippert 1966). The final pH of the medium was 7. The chemicals used for all the experiments were purchased from Sigma-Aldrich (St. Louis, USA). Sterile conditions were maintained throughout the experiments, and all the stock solutions used were either sterile filtered or autoclaved.

Five bacterial strains were used in this study, out of which four were environmental isolates from compost samples, which were deposited in the German Collection of Microorganisms and Cell Cultures (DSMZ), namely *Klebsiella* sp. strain A (DSM 104483), *Pseudomonas* sp. strain B (DSM 104484), *Pseudomonas plecoglossicida* strain C (DSM 104485), and *Pseudomonas* sp. strain Sigma (DSM 104486). (These strains are hereafter referred to as isolates A, B, C, and Sigma, respectively. In addition, the strain *P. putida* KT2440 (DSM 6125), purchased from DSMZ, was used.

### Isolation of bacterial strains from compost

Mature compost samples from a waste management plant were kindly provided by Sysav, South Scania Waste Company (Malmö, Sweden). The vegetable compost sample used in this study was from 2013, obtained from a depth of around 20 cm and coarse-grained. The sample was slightly alkaline with a pH around 8.

One gram (wet weight) of compost was washed with 4 mL of sterile 0.8% (*w*/*v*) NaCl solution by vortexing thoroughly. This suspension was left to settle for 5 min, and 100 μL of the supernatant was used to inoculate the liquid enrichment cultures. Enrichment cultures were made in 50-mL sterile tubes with 10 mL of M9 medium, supplemented with 5% (*v*/*v*) of a filtered kraft pulping process stream from spruce (softwood), or birch (hardwood), as the only source of carbon and energy. These streams contain a mixture of acid-soluble lignin degradation products. The sugar content of the hardwood stream was around 2.4 g L^−1^ (only glucose was detected), whereas the softwood stream contained 2.1 g L^−1^ of glucose and 0.8 g L^−1^ of other monosaccharides (xylose or mannose). Other enrichment cultures were supplemented with 5 g L^−1^ technical kraft lignin from Sigma-Aldrich (St. Louis, USA). A non-inoculated control tube was prepared for each one of the enrichment conditions assayed. Enrichment tubes and controls were further incubated at 30 °C and 180 rpm orbital shaking for 6 days.

After incubation, the culture was streaked on M9 agar plates with the same carbon sources and concentrations used in the enrichment. Plates were incubated at 30 °C until bacterial growth was detected. Microbial biomass from the plates was restreaked on the same media until individual colonies were isolated.

### Taxonomic identification of bacterial strains

Smears of the isolated colonies were observed in the optical microscopy in order to check the purity of the cultures and the bacterial aspect of all isolates. Five milliliters of Lysogeny broth (LB) was inoculated with each one of the isolates and grown overnight at 30 °C with shaking. Cells were subsequently harvested and used for purification of genomic DNA with the GeneJET Genomic DNA Purification Kit from Thermo Fisher Scientific Baltics (Vilnius, Lithuania) and submitted to Eurofins Genomics (Ebersberg, Germany) for dideoxy chain-termination sequencing in an ABI 3730xl DNA analyzer from Applied Biosystems (Carlsbad, USA), using the same universal primers.

Genomic DNA was used as a template for the PCR amplification of the 16S ribosomal RNA gene region with the universal primers 27F and 1492R. PCRs were carried out with Phusion Hot Start II High-Fidelity DNA Polymerase (Thermo Scientific) according to the manufacturer’s manual. Briefly, reactions with 150 μL total volume were prepared with approximately 200 ng of the corresponding genomic DNA as template. The PCR program was carried out as follows: initial denaturation at 98 °C for 2 min, 35 cycles of denaturation at 98 °C for 10 s, annealing at 60 °C for 20 s and extension at 72 °C for 35 s, and a final extension at 72 °C for 10 min. Amplified DNA fragments of around 1.5 kb were purified with GeneJET PCR Purification Kit (Thermo Scientific) and submitted for sequencing with the same universal primers to Eurofins Genomics (Ebersberg, Germany).

16S rRNA sequences from environmental isolates were submitted to the European Nucleotide Archive (ENA), the accession code for each sequence is as follows: LT671911 (isolate A/DSM 104483), LT671912 (isolate B/DSM 104484), LT671913 (isolate C/DSM 104485), and LT671914 (isolate Sigma/DSM 104486). Retrieved sequences were submitted to three different taxonomic identification servers: BLAST (16S ribosomal RNA database), EzTaxon (http://www.ezbiocloud.net/eztaxon) (Kim 2012), and RDP-II SeqMatch (http://rdp.cme.msu.edu/seqmatch/seqmatch_intro.jsp) (Cole 2014).

### Shake flask fermentations

Initially, the glycerol stocks from −80 °C were streaked on LB plates to obtain single colonies. Seed cultures were prepared by inoculating a single colony from the LB plates in a liquid M9 medium with 10 g L^−1^ of d-glucose. After incubation in shake flask for 17 h at 28 °C with 170 rpm orbital shaking, cells were harvested, washed with sterile saline solution, and used for inoculation. Shake flask experiments were conducted in 250-mL flasks containing 30 mL M9 media supplemented with 5 mM vanillin, vanillate, 4-hydroxybenzoate, *p*-coumarate, benzoate, and ferulate, individually or in mixtures. The flasks were inoculated and maintained at 28 °C with a shaker speed of 170 rpm. Aliquots of the culture samples were withdrawn at different times for analysis. All experiments were performed in duplicate.

Culture growth was determined spectrophotometrically by OD_620_. After each sampling, cells in the aliquots were harvested by centrifugation at 12,300*g* for 5 min. The supernatant was filtered (0.2 μm pore size) and frozen at −20 °C before ultra high-performance liquid chromatography (UHPLC) analysis.

### Determination of rates and yields

The measured OD_620_ was converted into biomass by measuring the cell dry weight during cultivation. Biomass yield (expressed both in g(g)^−1^ and g(mmol)^−1^) was calculated from the phase plane plot of biomass formed and substrate consumed. Maximum specific growth rates (h^−1^) were calculated from the plot of natural logarithm of biomass in the culture against the time of cultivation. The specific uptake rate of the carbon substrate was determined using the formula

$$ {q}_{\mathrm{Substrate}}=\frac{\mu}{Y_{SX}} $$

where *q*_Substrate_ is the specific substrate uptake rate (mmol (g_CDW_ h)^−1^), *μ* is the specific growth rate, and *Y*_*SX*_ is the biomass yield coefficient (g_CDW_(g)^−1^ or g_CDW_(mmol)^−1^).

### UHPLC analysis

A Waters Acquity UPLC system (Milford, USA) with a photodiode array detector was used for analysis. An Acquity UPLC BEH C_18_ column (Milford, USA) (100 × 2.1 mm, internal diameter) with a particle size of 1.7 μm was used. The temperature of the column was maintained at 47 °C. The eluents used were the binary system phases with A (95% water, 3% acetonitrile, and 2% acetic acid) and B (13% water, 85% acetonitrile, and 2% acetic acid) at the flow rate of 0.7 mL min^−1^. The method used for these analyses was adjusted as described in Schwarz et al. (2009). The aromatic compounds detected were quantified according to the area under the respective peaks against the calibrated standards.

## Results

### Isolation of bacterial strains from compost

**Fig. 1:**
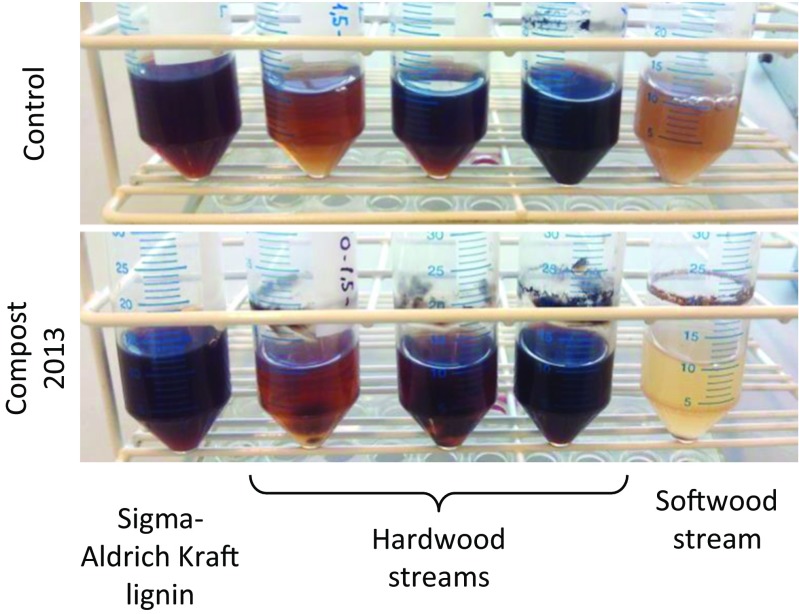
Enrichment cultures in the M9 medium supplemented with different sources of lignin-related products (indicated *below* the picture) after 6 days of incubation. Tubes in the *bottom panel* are inoculated with a mature compost wash; tubes in the *upper part* are non-inoculated controls

Several enrichment cultures with different lignin-related products as the sole carbon source were inoculated with extracts of mature compost samples and subsequently incubated for 6 days at 30 °C. In all the cultures enriched with hardwood waste streams from kraft pulping, a vigorous growth of filamentous fungi was observed, forming abundant mycelia which were suspended in the liquid medium, floating on the surface or adhered to the tube walls. In the cultures supplemented with softwood streams, by contrast, no fungal growth was observed, but a brownish biofilm was adhered to the wall of the tube. A notable discoloration of the medium was also seen when comparing inoculated tubes to the corresponding control tube (Fig. 1). In the case of enrichment culture with technical kraft lignin, some precipitate was observed, as well as a slight darkening of the medium compared to the control.

All enrichment cultures showing suspension growth or biofilms with bacterial appearance were streaked and incubated on agar plates with the same corresponding medium consecutively, until isolation of individual colonies was possible. Filamentous fungi were not within the scope of this work and were not cultured any further. Three isolates coming from softwood enrichment cultures (named A, B, and C), and one isolate from kraft lignin (named Sigma), were subjected to taxonomic identification, after verifying that all of them showed bacterial morphology under the optical microscope.

### Taxonomic identification of bacterial strains

Genomic DNA of the four bacterial isolates was isolated and used as template for the PCR amplification of the 16S rRNA genes. These amplified DNA fragments were further purified and sequenced with the universal primers 27F and 1492R. The retrieved sequences (Supplementary Table S1) were assembled and submitted to three different servers for taxonomic analysis. The candidate species with the highest score in each case are shown in Table 1.

**Table 1:** Taxonomic identification of bacterial isolates from compost according to 16S rRNA sequences. The highest score candidates are shown

Isolates	BLAST	Identity (%)	EzTaxon	Similarity (%)	RDP-II	Score
A	*Klebsiella pneumoniae* DSM 30104	99	*Klebsiella variicola* DSM 15968	99.79	*Klebsiella* sp. WR20	0.998
	*Klebsiella variicola* F2R9	99	*Klebsiella pneumoniae* DSM 30104	99.58	*Klebsiella variicola* GL6	0.997
	*Klebsiella pneumoniae* ATCC 13883	99	*Klebsiella quasipneumoniae* 01A030	99.57	*Klebsiella variicola* XF7	0.997
B	*Pseudomonas plecoglossicida* NBRC 103162	99	*Pseudomonas monteilii* NBRC 103158	99.79	*Pseudomonas putida* IARI-RP28	1.000
	*Pseudomonas taiwanensis* BRCR 17751	99	*Pseudomonas plecoglossicida* NBRC 103162	99.79	*Pseudomonas mosselii* L27	1.000
	*Pseudomonas monteilii* CIP 104883	99	*Pseudomonas taiwanensis* BRCR 17751	99.79	*Pseudomonas plecoglossicida* R4	1.000
C	*Pseudomonas plecoglossicida* NBRC 103162	99	*Pseudomonas plecoglossicida* NBRC 103162	100	*Pseudomonas plecoglossicida* P-9	0.998
	*Pseudomonas plecoglossicida* FPC951	99	*Pseudomonas monteilii* NBRC 103158	99.86	*Pseudomonas putida* TP0701	0.997
	*Pseudomonas taiwanensis* BCRC 17751	99	*Pseudomonas taiwanensis* BCRC 17751	99.86	*Pseudomonas monteilii* SB 3067	0.996
Sigma	*Pseudomonas chengduensis* MBR	99	*Pseudomonas alcaliphila* AL15–21	99.83	*Pseudomonas mendocina* PC6	0.994
	*Pseudomonas alcaliphila* NBRC 102411	99	*Pseudomonas chengduensis* MBR	99.83	*Pseudomonas toyotomiensis* SW237	0.994
	*Pseudomonas oleovorans* subsp. *lubricantis* RS1	99	*Pseudomonas toyotomiensis* HT-3	99.83	*Pseudomonas pseudoalcaligenes* C70b	0.994

### Growth characterization of *P. putida* KT2440 on lignin model compounds

There was a clear dominance of *Pseudomonas* spp. among the organisms showing the highest score in the 16S rRNA sequence analysis similarity search (Table 1). Hence, one of the best known *Pseudomonas* strains, which is *P. putida* KT2440, was obtained from culture collection, and its growth on six selected aromatic model compounds (vanillin, vanillate, 4-hydroxybenzoate, *p*-coumarate, benzoate, and ferulate) was assessed.

**Fig. 2:**
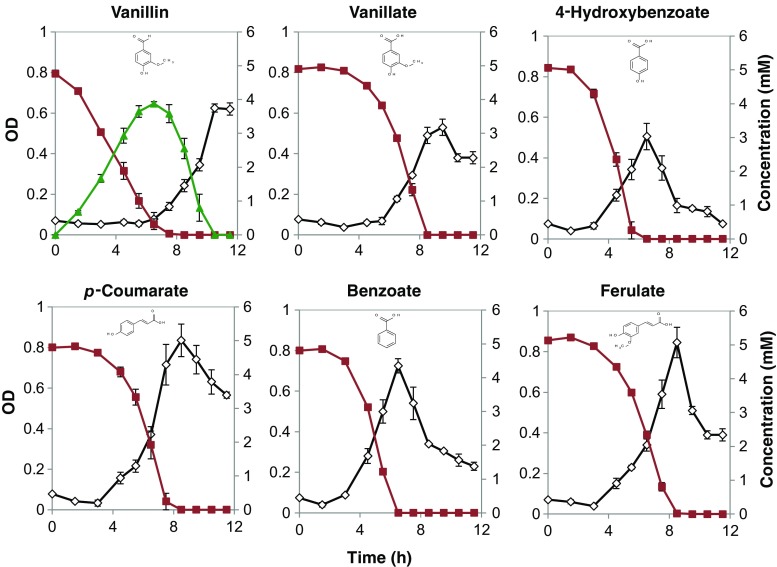
Growth of *P. putida* KT2440 on six selected lignin model compounds as the only carbon source in the M9 medium. Duplicate experiments were performed, and the standard deviations are shown with an *error bar*. OD and the concentrations of model compounds are indicated with a *black diamond* and *red square*, respectively. The curve indicated with *green triangle* is the concentration of vanillate

*P. putida* KT2440 was able to take up all the six aromatic compounds tested (Fig. 2). There was no appreciable growth on vanillin, but growth instead occurred in a second phase on the vanillate produced from vanillin (Fig. 2). The biomass yields were in the range of 0.06 to 0.074 g mmol^−1^ for the compounds, i.e., within the expected range for aerobic growth (the biomass yield for glucose was 0.06 g mmol^−1^). The highest specific growth rate, 0.27 h^−1^, was obtained for growth on benzoate, whereas the lowest growth rate, 0.21 h^−1^, was obtained on ferulate (Table 2). The specific rate on glucose, as a comparison, was determined to be 0.45 h^−1^ in the defined M9 medium used. The specific conversion rate of vanillin to vanillate was notably higher than the conversion rate of the carbon sources allowing growth. Another observation was that the biomass concentration started to decline as soon as the carbon source was depleted, without any apparent stationary phase; i.e., there seemed to be a need for an energy source to maintain biomass (Fig. 2).

**Table 2:** Specific growth rates and uptake rates of model compounds by *P. putida* KT2440

Compounds	Specific growth rate (h^−1^)	Uptake rates (mmol (g_CDW_ h)^−1^)	Yield (g(g^−1^))	Yield (g(mmol^−1^))
Vanillin	–	4.87 ± 0.04	–	–
Vanillate	0.22 ± 0.007	3.76 ± 0.15	0.357 ± 0.024	0.060 ± 0.004
Benzoate	0.27 ± 0.004	3.83 ± 0.04	0.581 ± 0.017	0.071 ± 0.002
*p*-Coumarate	0.26 ± 0.002	4.04 ± 0.37	0.396 ± 0.030	0.065 ± 0.005
4-HBA	0.26 ± 0.031	4.57 ± 0.38	0.435 ± 0.029	0.060 ± 0.004
Ferulate	0.21 ± 0.019	2.91 ± 0.27	0.381 ± 0.005	0.074 ± 0.001
Glucose	0.45 ± 0.050	7.44 ± 0.28	0.333 ± 0.028	0.060 ± 0.005

When vanillin was provided as the sole carbon substrate to *P. putida* KT2440, it was taken up at a rate of 4.87 mmol (g_CDW_ h)^−1^ and was mainly converted into vanillate (Fig. 2). The consumption rate of vanillate (3.76 mmol (g_CDW_ h)^−1^) was slower than that of vanillin, which means that the production of vanillate is faster than its consumption, resulting in a net accumulation. However, the maximum detected concentration of vanillate in this experiment was lower than what would be reached in a fully quantitative conversion of vanillin, which indicates a partially simultaneous conversion. Even so, there seemed to be very little growth until complete conversion of vanillin was achieved.

### Growth of isolated organisms and *P. putida* KT2440 on mixtures of model compounds

**Fig. 3:**
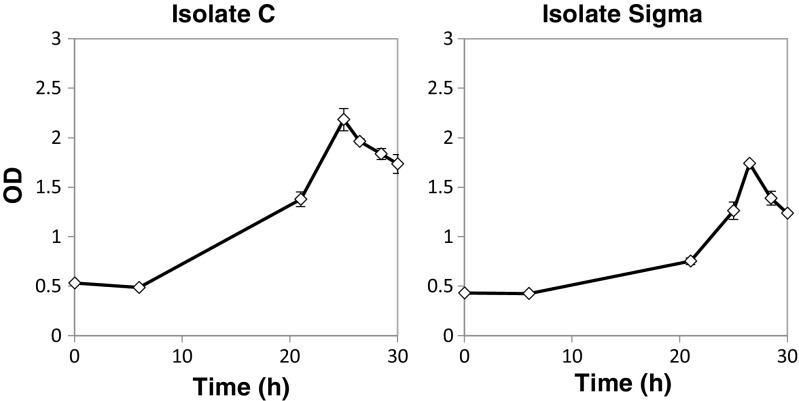
Growth of isolates C and Sigma on the mixture of vanillin, vanillate, and 4-hydroxybenzoate. OD is indicated with a *black diamond line*. The uptake of model compounds by these microorganisms was not measured as there was a lag phase (6 h) in their growth curves compared to isolates B and *P. putida* KT2440 (see Fig. 4)

**Fig. 4:**
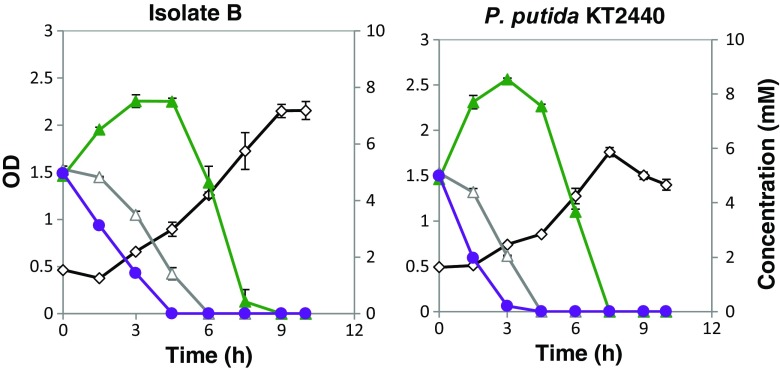
Growth of isolates B and *P. putida* KT2440 on the mixture of vanillin, vanillate, and 4-hydroxybenzoate. OD is indicated with a *black diamond line*. The concentration of model compounds is shown in *violet circle* (vanillin), *gray open triangle* (4-HBA), and *green closed triangles* (vanillate)

Three *Pseudomonas* spp. isolates (B, C and Sigma) along with *P. putida* KT2440 were selected for further characterization experiments on mixtures of lignin model compounds. Isolate A was likely a *Klebsiella* strain and was not further investigated for the present study (cf. Table 1). Shake flask experiments were carried out with 5 mM each of vanillin, vanillate, and 4-hydroxybenzoate as carbon sources in the M9 medium using an initial OD around 0.5. Two isolates (C and Sigma) showed an appreciable lag phase of at least 6 h (Fig. 3), and the maximum biomass concentration was not attained until after about 25 h; hence, these isolates were not further investigated. In contrast, the strain *P. putida* KT2440 and isolate B grew without any appreciable lag phase and the maximum biomass concentration was attained in less than 10 h. The substrate consumption rates for these fast-growing strains were measured, finding a complete assimilation of all model compounds within 10 h (Fig. 4).

Initially, vanillin was rapidly converted into vanillate. As pointed out above, there may have been partly a simultaneous uptake of vanillate and vanillin. After full conversion of vanillin, there was a net consumption of vanillate. The consumption of vanillin and vanillate was slightly faster by *P. putida* KT2440 than isolate B, apparently, but the difference was small. The uptake of 4-hydroxybenzoate began later than that of vanillin, but before complete conversion of vanillin. Uptake of 4-hydroxybenzoate and vanillate occurred simultaneously (Fig. 4).

**Fig. 5:**
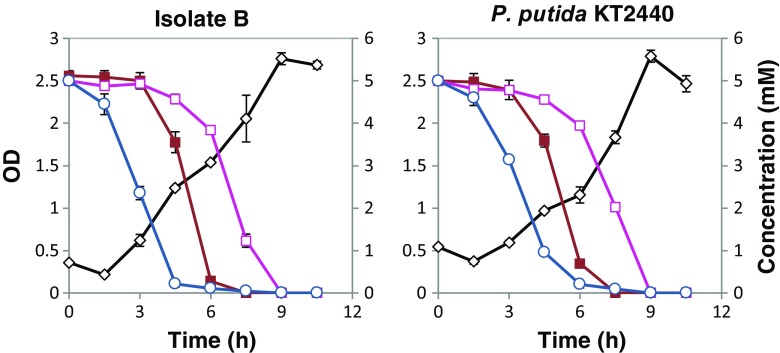
Growth of isolates B and *P. putida* KT2440 on the mixture of benzoate, *p*-coumarate, and ferulate. OD is indicated with a *black diamond line*. The concentration of model compounds is shown in *blue circle* (benzoic acid), *red closed square* (*p*-coumarate), and *pink open square* (ferulate)

Another set of growth experiments was made with a mixture of ferulate, *p*-coumarate, and benzoate (5 mM of each) with isolates B and *P. putida* KT2440, but not with isolates C and Sigma, due to their slow growth, as mentioned above. Again, isolates B and *P. putida* KT2440 showed similar growth curves, attaining maximum biomass concentration and consuming all the available substrates within the first 10 h (Fig. 5). Initially, benzoate was consumed, followed by *p*-coumarate and, finally, ferulate. There was some overlap between the consumptions of benzoate and *p*-coumarate, as well as between *p*-coumarate and ferulate, but practically, no simultaneous consumption of benzoate and ferulate.

**Fig. 6:**
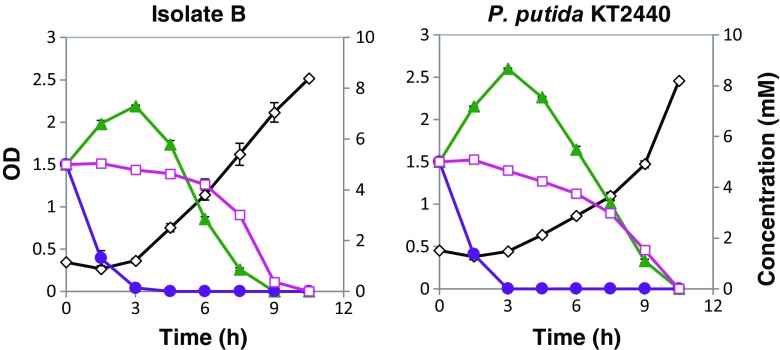
Growth of isolates B and *P. putida* KT2440 on the mixture of vanillin, vanillate, and ferulate. OD is indicated with a *black diamond line*. The concentration of model compounds is shown in *violet circle* (vanillin), *pink square* (ferulate), and *green triangle* (vanillate)

**Fig. 7:**
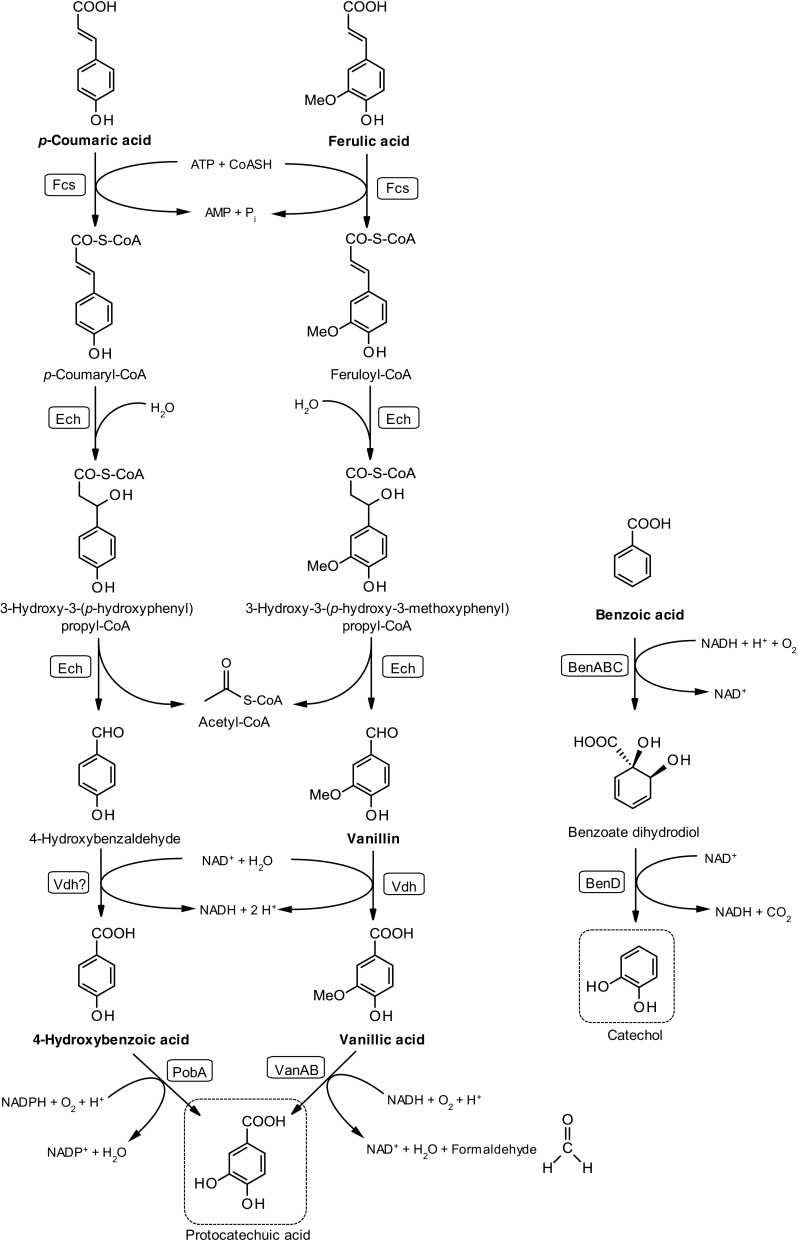
Main upper degradation pathways of ferulic acid, *p*-coumaric acid, and benzoates in *Pseudomonas putida* KT2440. The six model compounds used in this study are indicated in *bold*; names of the enzymes involved in each step of the pathway are shown in *boxes*. *Fcs* feruloyl-CoA synthase, *Ech* enoyl-CoA hydratase/lyase, *Vdh* vanillin dehydrogenase, *PobA* 4-hydroxybenzoate hydroxylase, *VanAB* vanillate *O*-demethylase complex, *BenABC* benzoate dioxygenase complex, *BenD* 1,6-dihydroxycyclohexa-2,4-diene-1-carboxylate dehydrogenase. Metabolic nodes that can undergo ring cleavage are shown in *dashed boxes*

Further experiments were carried out with the three model compounds from the same branch of the upper funneling pathway (cf. Fig. 7). Growth of isolates B and *P. putida* KT2440 was analyzed with 5 mM each of vanillin, vanillate, and ferulate (Fig. 6). As expected, vanillin was converted first. After the conversion of vanillin, vanillate was taken in, while ferulate was taken up last. There seems to be a slight difference between isolates B and *P. putida* KT2440—also here—with a somewhat more quantitative conversion of vanillin into vanillate by *P. putida* KT2440. This could be a result of a faster uptake of vanillate by isolate B.

## Discussion

This work was aimed at detecting and characterizing robust and easily culturable bacteria from compost, displaying an enzymatic repertoire which allows them to grow using lignin-related aromatic compounds. Mature compost from urban vegetable waste was chosen as a source of microorganisms because of its composition and being very heterogeneous and certainly enriched in polymeric aromatic complexes, derived ultimately from plant lignocellulose. A small-scale growth-based screening was carried out to this end, in which several aromatic compound sources were employed for the preparation of enrichment media. One of these sources was a technical lignin produced by kraft pulping, whereas the others were waste streams also from a kraft process, specifically coming from birch (hardwood) or spruce (softwood). These filtrated streams are known to contain a high diversity of lignin degradation products, with different molecular size range, solubilized as a result of the cooking process with NaOH and Na_2_S (Chakar and Ragauskas 2004). They also contain variable amounts of carbohydrates, generally low, since separation of lignin from the carbohydrates is the main goal of the kraft pulping process. Technical kraft lignin was also used in order to identify microorganisms able to metabolize polymeric lignin with higher molecular size.

The most predominant lignin-degrading microorganisms in most natural aerobic environments are fungi. With regard to bacterial lignin degradation, most of the species described so far fall into the classes *Actinobacteria* and α- or γ-*Proteobacteria* (Bugg et al. 2011b; Taylor et al. 2012), with a few notable exceptions from the class *Bacilli*, e.g., the genus *Bacillus* or *Aneurinibacillus* (Raj et al. 2007). In terms of metabolism of aromatic compounds, the diversity of organisms and strategies is considerably higher, including numerous species from the classes β- and δ-*Proteobacteria*, notably the order *Burkholderiales* (Pérez-Pantoja et al. 2012), as well as *Bacilli* (Fuchs et al. 2011). The bacterial isolates identified with this screening agree with the expected results, since all the bacteria with the highest score belong to the γ-*Proteobacteria* class, three of them being species of the *Pseudomonas* genus, and one of them named isolate A, being an enterobacterium of the genus *Klebsiella*. Regarding the latter, some isolates of *Klebsiella* have been reported to exhibit a high tolerance to aromatic molecules or even lignin and aromatic compound-degrading capabilities (Martín et al. 1991; Nishikawa et al. 1988; Woo et al. 2014). As for the genus *Pseudomonas*, extensive and successful research has been conducted in the last decades in order to elucidate the complex network of metabolic pathways involved in the degradation of aromatic compounds by these bacteria. Especially the strain *P. putida* KT2440 has gained more attention recently due to its natural robustness and stress endurance (Nikel et al. 2014), which led us to choose it as a model strain for an in-depth study in this contribution.

*P. putida* is a ubiquitous Gram-negative soil bacterium, which has been widely studied for its remarkable substrate-utilizing capacity (Jiménez et al. 2002; Wackett 2003). Initially, due to its metabolic versatility, these bacteria were presumed to be significant for bioremediation (de Lorenzo 2008). Currently, *P. putida* is getting increasing attention in industrial biotechnology for the synthesis of bio-based polymers and fine chemicals (Poblete-Castro et al. 2012). The strain *P. putida* KT2440 that utilizes a broad range of aromatic compounds shows an outstanding tolerance to environmental stress and serves as a host to produce targeted molecules. The generally recognized as safe (GRAS) status of the organism, together with its completely annotated genome sequence (Belda et al. 2016; Nelson et al. 2002), facilitates its use for industrial applications. This strain has been successfully domesticated using synthetic biology (Martínez-García et al. 2014; Nikel et al. 2014; Puchalka et al. 2008). Recently, *P. putida* has gained importance towards the breakdown of lignin-related aromatic compounds. It has a specialized β-ketoadipate pathway for the utilization of monomeric compounds derived from lignin degradation, as the sole source of carbon and energy (Kim et al. 2006). There have been several proteomic studies to confirm the aromatic catabolic pathways in *P. putida* KT2440 using monocyclic aromatic compounds such as *p*-hydroxybenzoate, vanillin, and benzoate (Kim et al. 2006). Under nitrogen-limiting conditions, it can utilize the lignin from alkaline-pretreated liquor and accumulate polyhydroxyalkanoates (Linger et al. 2014). Vardon et al. (2015), demonstrating the production of *cis*,*cis*-muconate from *p*-coumarate, ferulate, and benzoate in an engineered strain of *P. putida* KT2440. β-Ketoadipic acid and muconolactone were produced from protocatechuic acid by the deactivation of PcaJ (a β-ketoadipate/succinyl-CoA transferase) in *P. putida* KT2440 (Okamura-Abe et al. 2016). Also, to increase the yield of pyruvate production from aromatic molecules, the catechol *ortho* degradation pathway in *P. putida* KT2440 was replaced with a *meta*-cleavage pathway from *P. putida* mt-2. This study encapsulates how to yield the desired products from lignin-related compounds by tuning the aromatic degradation pathways in *P. putida* (Johnson and Beckham 2015).

Despite the large amount of investigations developed with *P. putida* with respect to its aromatic metabolism, some important parameters, such as the kinetics and flux capacities of the lignin model compounds via the β-ketoadipate pathway, are not well studied. Hence, this study focuses on the kinetics of six selected lignin model compounds in *P. putida* KT2440. Ferulate, vanillin, and vanillate belong to the coniferyl alcohol branch of the upper funneling pathway, while 4-hydroxybenzoate and *p*-coumarate are from the coumaryl branch; the compounds featured in both branches only differ in the methoxylation in position 3 of the benzene ring (Fig. 7). Both of these coniferyl and coumaryl branches converge to protocatechuate and follow the β-ketoadipate pathway. Benzoate is catabolized by *P. putida* through the catechol branch of the β-ketoadipate pathway (Harwood et al. 1994). This pathway proceeds with an *ortho* ring cleavage, yielding succinate and acetyl-CoA as the final products, which will enter the tricarboxylic acid (TCA) cycle to promote growth of the organism. The specific growth rate of *P. putida* KT2440 on glucose in the present study was 0.45 h^−1^ (Table 2) which is in good agreement (0.48 h^−1^) with the results reported by Sun et al. (2007). The growth rates on 4-hydroxybenzoate and *p*-coumarate were higher than those of the compounds from the coniferyl branch; this fact shows that the coumaryl branch is faster (Table 2). This again confirmed the slower uptake rates of the compounds from the coniferyl branch of the upper funneling pathway. This can be due to the presence of a phenylmethyl ether linkage in the compounds of the coniferyl branch, which is a unique linkage of lignin structure, and thus, it is a critical factor determining the catabolism rate of these compounds (Okamura-Abe et al. 2016). Notably, the uptake rate of ferulate, 2.91 mmol (g_CDW_ h)^−1^, was the lowest of the tested compounds, and in agreement with this, the specific growth on ferulate was also the lowest (0.21 h^−1^). The uptake rate of benzoic acid was 3.83 mmol (g_CDW_ h)^−1^, in agreement with the reported value of 3.4 mmol (g_CDW_ h)^−1^ by Sudarsan et al. (2016).

When vanillin was provided as the sole carbon source, *P. putida* KT2440 takes it up at a rate of 4.87 mmol (g_CDW_ h)^−1^ and converts it into vanillate. Since the consumption rate of vanillate is slower than that of vanillin, this leads to an accumulation of vanillate in the medium, which is subsequently assimilated. The rapid bioconversion of the toxic aldehyde vanillin to a less toxic compound—here vanillate—is similar to what has been observed in other organisms. Vanillin is a potent inhibitor to many other organisms, such as *Saccharomyces cerevisiae*. In *S. cerevisiae*, vanillin is reduced to the less toxic vanillyl alcohol by NADPH-dependent alcohol dehydrogenases (Nguyen et al. 2015). Vanillin was also identified as the most toxic lignocellulosic compound for the yeast *Kluyveromyces marxianus* and was reported to reduce cell growth and fermentation by 90% (Oliva et al. 2004). Previous studies showed that there were major reorganizations of about 662 proteins in the *P. putida* KT2440 proteome in response to vanillin. The most notable changes were the increased expression of vanillin-degrading enzymes in the central metabolic pathway followed by changes in the expression of certain transporters (Simon et al. 2014). It has also been found that Vdh vanillin dehydrogenase (NCBI accession number NP_745497.1) is not the only enzyme involved, but there are several other aldehyde dehydrogenases that are activated in response to vanillin. There was almost no vanillate excretion when ferulate was used as a carbon source, even though ferulate has to be catabolized via vanillate. This result points out that the uptake rate of ferulate is even lower than that of vanillate.

The growth curves for the mixture of model compounds seems to reflect diauxic growth in some cases (Figs. 4 and 5). Three model compounds were mixed at a time in a systematic way: (i) two compounds from the same funneling branch and one from a different branch, (ii) all three compounds from the same branch, and (iii) three compounds, each from a different branch. Vanillin, 4-hydroxybenzoate, and benzoate were consumed first in the mixtures (Figs. 4, 5, and 6). Vanillin is likely converted first because of its toxicity, whereas 4-hydroxybenzoate and benzoate are easier to consume compared to the compounds from the coniferyl branch. Ferulate was always the least preferred of the substrates.

As illustrated in this work, *P. putida* has an excellent metabolic capability to funnel various lignin-related compounds via the β-ketoadipate pathway. The conversion rates of these compounds were approximately half of the values for glucose. The main bottleneck appears to be the removal of the phenylmethyl ether linkage, which is a typical structure of lignin.

It is important to point out that the technical lignin as such or the depolymerized lignin represents much more complex and challenging substrates than selected model compounds. Understanding the metabolism of these more complex mixtures by pseudomonads is a necessary next step for real valorization of lignin.

## Electronic supplementary material

ESM 1 (PDF 297 kb).
