# Supplementary material for: Conversion of lignin model compounds by Pseudomonas putida KT2440 and isolates from compost
Source: Appl Microbiol Biotechnol. 2017 Mar 15;101(12):5059–70. doi: 10.1007/s00253-017-8211-y (PMC5486835; doi:10.1007/s00253-017-8211-y)
Supplement: Supplementary file 1 — (PDF 297 kb). [file 253_2017_8211_MOESM1_ESM.pdf]

Supplementary materials

Journal: Applied Microbiology and Biotechnology

Research article title: Conversion of lignin model compounds by *Pseudomonas putida* KT2440 and isolates from compost

Authors: Krithika Ravi[1], Javier García-Hidalgo[2*], Marie F Gorwa-Grauslund[2], and Gunnar Lidén[1]

Affiliations:

[1]Department of Chemical Engineering, Lund University, P.O. Box 124, SE-221 00 Lund, Sweden

[2]Department of Chemistry, Applied Microbiology, Lund University, P.O. Box 124, SE-221 00 Lund, Sweden

Contact:

[*]Corresponding author: javier.garcia_hidalgo@tmb.lth.se Phone number: +46 462228328

Table S1: 16S rRNA gene sequences of the bacterial isolates detected in this study. Sequences were obtained with the forward and reverse PCR primers 27F and 1492R, and manually assembled into the complete sequences.

Isolate A

1	ACCTGCAAGT	CGAGCGGTAG	CACAGAGAGC	TTGCTCTCGG	GTGACGAGCG	GCGGACGGGT
61	GAGTAATGTC	TGGGAAACTG	CCTGATGGAG	GGGGATAACT	ACTGGAAACG	GTAGCTAATA
121	CCGCATAACG	TCGCAAGACC	AAAGTGGGGG	ACCTTCGGGC	CTCATGCCAT	CAGATGTGCC
181	CAGATGGGAT	TAGCTGGTAG	GTGGGGTAAC	GGCTCACCTA	GGCGACGATC	CCTAGCTGGT
241	CTGAGAGGAT	GACCAGCCAC	ACTGGAACTG	AGACACGGTC	CAGACTCCTA	CGGGAGGCAG
301	CAGTGGGGAA	TATTGCACAA	TGGGCGCAAG	CCTGATGCAG	CCATGCCGCG	TGTGTGAAGA
361	AGGCCTTCGG	GTTGTAAAGC	ACTTTCAGCG	GGGAGGAAGG	CGATGAGGTT	AATAACCTTG
421	TCGATTGACG	TTACCCGCAG	AAGAAGCACC	GGCTAACTCC	GTGCCAGCAG	CCGCGGTAAT
481	ACGGAGGGTG	CAAGCGTTAA	TCGGAATTAC	TGGGCGTAAA	GCGCACGCAG	GCGGTCTGTC
541	AAGTCGGATG	TGAAATCCCC	GGGCTCAACC	TGGGAACTGC	ATTCGAAACT	GGCAGGCTAG
601	AGTCTTGTAG	AGGGGGGTAG	AATTCCAGGT	GTAGCGGTGA	AATGCGTAGA	GATCTGGAGG
661	AATACCGGTG	GCGAAGGCGG	CCCCCTGGAC	AAAGACTGAC	GCTCAGGTGC	GAAAGCGTGG
721	GGAGCAAACA	GGATTAGATA	CCCTGGTAGT	CCACGCTGTA	AACGATGTCG	ATTTGGAGGT
781	TGTGCCCTTG	AGGCGTGGCT	TCCGGAGCTA	ACGCGTTAAA	TCGACCGCCT	GGGGAGTACG
841	GCCGCAAGGT	TAAAACTCAA	ATGAATTGAC	GGGGGCCCGC	ACAAGCGGTG	GAGCATGTGG
901	TTTAATTCGA	TGCAACGCGA	AGAACCTTAC	CTGGTCTTGA	CATCCACAGA	ACTTTCCAGA
961	GATGGATTGG	TGCCTTCGGG	AACTGTGAGA	CAGGTGCTGC	ATGGCTGTCG	TCAGCTCGTG
1021	TTGTGAAATG	TTGGGTTAAG	TCCCGCAACG	AGCGCAACCC	TTATCCTTTG	TTGCCAGCGG
1081	TTAGGCCGGG	AACTCAAAGG	AGACTGCCAG	TGATAAACTG	GAGGAAGGTG	GGGATGACGT
1141	CAAGTCATCA	TGGCCCTTAC	GACCAGGGCT	ACACACGTGC	TACAATGGCA	TATACAAAGA
1201	GAAGCGACCT	CGCGAGAGCA	AGCGGACCTC	ATAAAGTATG	TCGTAGTCCG	GATTGGAGTC
1261	TGCAACTCGA	CTCCATGAAG	TCGGAATCGC	TAGTAATCGT	AGATCAGAAT	GCTACGGTGA
1321	ATACGTTCCC	GGGCCTTGTA	CACACCGCCC	GTCACACCAT	GGGAGTGGGT	TGCAAAAGAA
1381	GTAGGTAGCT	TAACCTTCGG	GAGGGCGCTA	CCA		

Isolate B

1	ACCATGCAAG	TCGAGCGGAT	GACGGGAGCT	TGCTCCTTGA	TTCAGCGGCG	GACGGGTGAG
61	TAATGCCTAG	GAATCTGCCT	GGTAGTGGGG	GACAACGTTT	CGAAAGGAAC	GCTAATACCG
121	CATACGTCCT	ACGGGAGAAA	GCAGGGGACC	TTCGGGCCTT	GCGCTATCAG	ATGAGCCTAG
181	GTCGGATTAG	CTAGTTGGTG	GGGTAATGGC	TCACCAAGGC	GACGATCCGT	AACTGGTCTG
241	AGAGGATGAT	CAGTCACACT	GGAACTGAGA	CACGGTCCAG	ACTCCTACGG	GAGGCAGCAG
301	TGGGGAATAT	TGGACAATGG	GCGAAAGCCT	GATCCAGCCA	TGCCGCGTGT	GTGAAGAAGG
361	TCTTCGGATT	GTAAAGCACT	TTAAGTTGGG	AGGAAGGGCA	GTAAGTTAAT	ACCTTGCTGT

421 TTTGACGTTA CCGACAGAAT AAGCACCGGC TAACTCTGTG CCAGCAGCCG CGGTAATACA
481 GAGGGTGCAA GCGTTAATCG GAATTACTGG GCGTAAAGCG CGCGTAGGTG GTTTGTTAAG
541 TTGGATGTGA AAGCCCCGGG CTCAACCTGG GAACTGCATC CAAAACTGGC AAGCTAGAGT
601 ACGGTAGAGG GTGGTGGAAT TTCCTGTGTA GCGGTGAAAT GCGTAGATAT AGGAAGGAAC
661 ACCAGTGGCG AAGGCGACCA CCTGGACTGA TACTGACACT GAGGTGCGAA AGCGTGGGGA
721 GCAAACAGGA TTAGATACCC TGGTAGTCCA CGCCGTAAAC GATGTCAACT AGCCGTTGGA
781 ATCCTTGAGA TTTTAGTGGC GCAGCTAACG CATTAAGTTG ACCGCCTGGG GAGTACGGCC
841 GCAAGGTTAA AACTCAAATG AATTGACGGG GGCCCGCACA AGCGGTGGAG CATGTGGTTT
901 AATTCGAAGC AACGCGAAGA ACCTTACCAG GCCTTGACAT GCAGAGAACT TTCCAGAGAT
961 GGATTGGTGC CTTCGGGAAC TCTGACACAG GTGCTGCATG GCTGTCGTCA GCTCGTGTCG
1021 TGAGATGTTG GGTTAAGTCC CGTAACGAGC GCAACCCTTG TCCTTAGTTA CCAGCACGTT
1081 ATGGTGGGCA CTCTAAGGAG ACTGCCGGTG ACAAACCGGA GGAAGGTGGG GATGACGTCA
1141 AGTCATCATG GCCCTTACGG CCTGGGCTAC ACACGTGCTA CAATGGTCGG TACAGAGGGT
1201 TGCCAAGCCG CGAGGTGGAG CTAATCTCAC AAAACCGATC GTAGTCCGGA TCGCAGTCTG
1261 CAACTCGACT GCGTGAAGTC GGAATCGCTA GTAATCGCGA ATCAGAATGT CGCGGTGAAT
1321 ACGTTCCCGG GCCTTGTACA CACCGCCCGT CACACCATGG GAGTGGGTTG CACCAGAAGT
1381 AGCTAGTCTA ACCTTCGGGA GGA

Isolate C

1 ACCATGCAAG TCGAGCGGAT GACGGGAGCT TGCTCCTTGA TTCAGCGGCG GACGGGTGAG
61 TAATGCCTAG GAATCTGCCT GGTAGTGGGG GACAACGTTT CGAAAGGAAC GCTAATACCG
121 CATACGTCCT ACGGGAGAAA GCAGGGGACC TTCGGGCCTT GCGCTATCAG ATGAGCCTAG
181 GTCGGATTAG CTAGTTGGTG GGGTAATGGC TCACCAAGGC GACGATCCGT AACTGGTCTG
241 AGAGGATGAT CAGTCACACT GGAACTGAGA CACGGTCCAG ACTCCTACGG GAGGCAGCAG
301 TGGGGAATAT TGGACAATGG GCGAAAGCCT GATCCAGCCA TGCCGCGTGT GTGAAGAAGG
361 TCTTCGGATT GTAAAGCACT TTAAGTTGGG AGGAAGGGCA GTAAGCTAAT ACCTTGCTGT
421 TTTGACGTTA CCGACAGAAT AAGCACCGGC TAACTCTGTG CCAGCAGCCG CGGTAATACA
481 GAGGGTGCAA GCGTTAATCG GAATTACTGG GCGTAAAGCG CGCGTAGGTG GTTCGTTAAG
541 TTGGATGTGA AAGCCCCGGG CTCAACCTGG GAACTGCATC CAAAACTGGC GAGCTAGAGT
601 ACGGTAGAGG GTGGTGGAAT TTCCTGTGTA GCGGTGAAAT GCGTAGATAT AGGAAGGAAC
661 ACCAGTGGCG AAGGCGACCA CCTGGACTGA TACTGACACT GAGGTGCGAA AGCGTGGGGA
721 GCAAACAGGA TTAGATACCC TGGTAGTCCA CGCCGTAAAC GATGTCAACT AGCCGTTGGA
781 ATCCTTGAGA TTTTAGTGGC GCAGCTAACG CATTAAGTTG ACCGCCTGGG GAGTACGGCC
841 GCAAGGTTAA AACTCAAATG AATTGACGGG GGCCCGCACA AGCGGTGGAG CATGTGGTTT
901 AATTCGAAGC AACGCGAAGA ACCTTACCAG GCCTTGACAT GCAGAGAACT TTCCAGAGAT
961 GGATTGGTGC CTTCGGGAAC TCTGACACAG GTGCTGCATG GCTGTCGTCA GCTCGTGTCG
1021 TGAGATGTTG GGTTAAGTCC CGTAACGAGC GCAACCCTTG TCCTTAGTTA CCAGCACGTT

1081 ATGGTGGGCA CTCTAAGGAG ACTGCCGGTG ACAAACCGGA GGAAGGTGGG GATGACGTCA

1141 AGTCATCATG GCCCTTACGG CCTGGGCTAC ACACGTGCTA CAATGGTCGG TACAGAGGGT

1201 TGCCAAGCCG CGAGGTGGAG CTAATCTCAC AAAACCGATC GTAGTCCGGA TCGCAGTCTG

1261 CAACTCGACT GCGTGAAGTC GGAATCGCTA GTAATCGCGA ATCAGAATGT CGCGGTGAAT

1321 ACGTTCCCGG GCCTTGTACA CACCGCCCGT CACACCATGG GAGTGGGTTG CACCAGAAGT

1381 AGCTAGTCTA ACCTTCGGGA GGACGGTACC

Isolate Sigma

1 ATCAGTCACA CTGGAACTGA GACACGGTCC AGACTCCTAC GGGAGGCAGC AGTGGGGAAT

61 ATTGGACAAT GGGCGAAAGC CTGATCCAGC CATGCCGCGT GTGTGAAGAA GGTCTTCGGA

121 TTGTAAAGCA CTTTAAGTTG GGAGGAAGGG CATTAACCTA ATACGTTAGT GTTTTGACGT

181 TACCGACAGA ATAAGCACCG GCTAACTTCG TGCCAGCAGC CGCGGTAATA CGAAGGGTGC

241 AAGCGTTAAT CGGAATTACT GGGCGTAAAG CGCGCGTAGG TGGTTCGTTA AGTTGGATGT

301 GAAAGCCCCG GGCTCAACCT GGGAACTGCA TCCAAAACTG GCGAGCTAGA GTACGGTAGA

361 GGGTGGTGGA ATTTCCTGTG TAGCGGTGAA ATGCGTAGAT ATAGGAAGGA ACACCAGTGG

421 CGAAGGCGAC CACCTGGACT GATACTGACA CTGAGGTGCG AAAGCGTGGG GAGCAAACAG

481 GATTAGATAC CCTGGTAGTC CACGCCGTAA ACGATGTCAA CTAGCCGTTG GGTTCCTTGA

541 GAACTTAGTG GCGCAGCTAA CGCATTAAGT TGACCGCCTG GGGAGTACGG CCGCAAGGTT

601 AAAACTCAAA TGAATTGACG GGGGCCCGCA CAAGCGGTGG AGCATGTGGT TTAATTCGAA

661 GCAACGCGAA GAACCTTACC TGGCCTTGAC ATGCTGAGAA CTTTCCAGAG ATGGATTGGT

721 GCCTTCGGGA ACTCAGACAC AGGTGCTGCA TGGCCGTCGT CAGCTCGTGT CGTGAGATGT

781 TGGGTTAAGT CCCGTAACGA GCGCAACCCT TGTCCTTAGT TACCAGCACG TAATGGTGGG

841 CACTCTAAGG AGACTGCCGG TGACAAACCG GAGGAAGGTG GGGATGACGT CAAGTCATCA

901 TGGCCCTTAC GGCCAGGGCT ACACACGTGC TACAATGGTC GGTACAAAGG GTTGCCAAGC

961 CGCGAGGTGG AGCTAATCCC ATAAAACCGA TCGTAGTCCG GATCGCAGTC TGCAACTCGA

1021 CTGCGTGAAG TCGGAATCGC TAGTAATCGT GAATCAGAAT GTCACGGTGA ATACGTTCCC

1081 GGGCCTTGTA CACACCGCCC GTCACACCAT GGGAGTGGGT TGCTCCAGAA GTAGCTAGTC

1141 TAACCTTCGG
